# Association of white matter hyperintensities and gray matter volume with cognition in older individuals without cognitive impairment

**DOI:** 10.1007/s00429-015-1034-7

**Published:** 2015-04-02

**Authors:** Zoe Arvanitakis, Debra A. Fleischman, Konstantinos Arfanakis, Sue E. Leurgans, Lisa L. Barnes, David A. Bennett

**Affiliations:** 1 Rush Alzheimer’s Disease Center, Rush University Medical Center, 600 S. Paulina Ave, Suite 1020, Chicago, IL 60612 USA; 2 Department of Neurological Sciences, Rush University Medical Center, Chicago, USA; 3 Department of Behavioral Sciences, Rush University Medical Center, Chicago, USA; 4 Department of Diagnostic Radiology and Nuclear Medicine, Rush University Medical Center, Chicago, USA; 5 Department of Biomedical Engineering, Illinois Institute of Technology, Rush University Medical Center, Chicago, USA

**Keywords:** Aging, Cognition, Brain, MRI, Volume, White matter hyperintensities, Gray matter, Voxel-wise analyses

## Abstract

Both presence of white matter hyperintensities (WMH) and smaller total gray matter volume on brain magnetic resonance imaging (MRI) are common findings in old age, and contribute to impaired cognition. We tested whether total WMH volume and gray matter volume had independent associations with cognition in community-dwelling individuals without dementia or mild cognitive impairment (MCI). We used data from participants of the Rush Memory and Aging Project. Brain MRI was available in 209 subjects without dementia or MCI (mean age 80; education = 15 years; 74 % women). WMH and gray matter were automatically segmented, and the total WMH and gray matter volumes were measured. Both MRI-derived measures were normalized by the intracranial volume. Cognitive data included composite measures of five different cognitive domains, based on 19 individual tests. Linear regression analyses, adjusted for age, sex, and education, were used to examine the relationship of logarithmically-transformed total WMH volume and of total gray matter volume to cognition. Larger total WMH volumes were associated with lower levels of perceptual speed (*p* < 0.001), but not with episodic memory, semantic memory, working memory, or visuospatial abilities (all *p* > 0.10). Smaller total gray matter volumes were associated with lower levels of perceptual speed (*p* = 0.013) and episodic memory (*p* = 0.001), but not with the other three cognitive domains (all *p* > 0.14). Larger total WMH volume was correlated with smaller total gray matter volume (*p* < 0.001). In a model with both MRI-derived measures included, the relation of WMH to perceptual speed remained significant (*p* < 0.001), while gray matter volumes were no longer related (*p* = 0.14). This study of older community-dwelling individuals without overt cognitive impairment suggests that the association of larger total WMH volume with lower perceptual speed is independent of total gray matter volume. These results help elucidate the pathological processes leading to lower cognitive function in aging.

## Introduction

White matter hyperintensities (WMH) are brain white matter lesions with high signal on *T*_2_-weighted fluid-attenuated inversion recovery (FLAIR) magnetic resonance imaging (MRI). These lesions are believed to represent underlying pathologic changes which are variable in nature and severity, including alterations in myelin and axon structure, gliosis, and small vessel disease (Gouw et al. 2011). Clinical experience and mounting research data suggest that WMH are very common in aging and associated with a range of vascular diseases including stroke (Breteler et al. 1994; Raz et al. 2012; Burton et al. 2004). The clinical significance of WMH has been well studied, and associations have been demonstrated with impaired neurologic function including cognitive and motor impairment, as well as with death (Kerber et al. 2006; Silbert et al. 2008; Debette and Markus 2010). Yet, despite better recognition of WMH with increased availability and use of sophisticated neuroimaging technology, and the consensus that WMH should no longer be considered an “incidental” finding on neuroimaging, little information is available on the relationship of WMH with neurologic function in individuals without overt neurologic impairment. The data to date suggest that WMH are likely associated with subtle changes in neurologic function, including lower cognitive function, even among healthy older individuals without overt cognitive impairment [e.g., without Alzheimer’s disease or mild cognitive impairment (MCI)], and these results have been supported by meta-analyses (DeCarli et al. 1995, Gunning-Dixon and Raz 2000; Nordahl et al. 2006; Ishikawa et al. 2012). However, few studies have systematically examined the relationship of WMH with a range of cognitive domains among older individuals without neurologic conditions or diseases, and results of these few studies are mixed, with data suggesting associations with some domains (e.g., executive function) but not others (Vannorsdall et al. 2009; Murray et al. 2010; Hedden et al. 2012).

While a body of literature supports a relationship of gray matter to cognition in older persons, including among persons without known cognitive impairment (Jack et al. 2000; Kramer et al. 2007; Fleischman et al. 2013), few reports are available that take the effects on cognition of both WMH and gray matter volumes into account simultaneously. Indeed, among studies examining white matter together with gray matter, many had small samples of cognitively normal subjects, were restricted to MRI volumes in select brain regions, or did not measure WMH lesions (Hedden et al. 2012; Mueller et al. 2010; Taki et al. 2011; Fletcher et al. 2013; Royle et al. 2013; Wirth et al. 2013; Papp et al. 2014). No study that we know of has directly tested independence of both total WMH and total gray matter volume effects on cognition among a large group of older individuals without cognitive impairment. Studies that consider multiple brain measures simultaneously are needed to improve understanding of the interactions of neuropathologic events and the pathophysiologic cascade. Further, studies of cognitively-normal individuals are critical to shed light on the earliest factors that may play a role in neurologic decline and, as such, are important for identifying plausible strategies for prevention of cognitive impairment, and, ultimately, for improving public health.

This study examines the relation of total WMH volume to cognitive function in different cognitive domains, and tests whether the relation is independent of total gray matter volume. We used brain MRI and neuropsychologic data from more than 200 well-characterized, older community-dwelling women and men without dementia or MCI, participating in an epidemiologic study of aging, the Rush Memory and Aging Project. In the first set of analyses, we examined the relation of total WMH volume on brain MRI scans to level of cognitive function in composite measures of five separate cognitive domains. Secondary analyses determined the relative contribution of WMH to cognition, compared to that of demographic variables, and addressed whether findings were modified by age. In the second set of analyses, we examined for independence of effects of total WMH volume and total gray matter volume on cognition.

## Methods

### Cohort

Individuals were enrolled in an ongoing epidemiologic, cohort study of aging, the Memory and Aging Project. Older community-dwelling individuals without known dementia were invited to participate in the study. All participants consented to annual detailed clinical evaluations. The study was approved by the Rush University Medical Center Institutional Review Board, and is funded by the National Institute on Aging. Detailed methods of the cohort study are found elsewhere (Bennett et al. 2012a). Briefly, the Memory and Aging Project began enrolling participants in 1997, and 1740 individuals had enrolled as of December 2013. Brain MRI data collection began in 2009, and more than 450 individuals have undergone a scan since that time. Analyses for this study were conducted among individuals with MRI images processed to date, with data available on both WMH and gray matter volumes, in which neither dementia nor MCI was present at the time of the brain scan (see below). While this is the first study reporting our WMH results, we have previously published structural MRI results (Fleischman et al. 2010, 2014; Bis et al. 2012; Stein et al. 2012; Arfanakis et al. 2013; Chauhan et al. 2015).

### Clinical evaluations

A uniform, structured, baseline clinical evaluation was administered to all subjects, including testing of cognitive function (see below). Annual follow-up evaluations were identical in all essential components. For this study, we used data from the clinical evaluation at the same follow-up cycle as the MRI scan (mean time interval between clinical evaluation and MRI scan 65.7 days, SD 57.8).

Cognitive function was evaluated using a battery of 21 individual neuropsychological tests, as previously published (Bennett et al. 2012a). The Mini-Mental State Examination (MMSE) was used to describe the cohort and complex ideational material was used for diagnostic classification. The other 19 tests were grouped to form composite scores in five different cognitive domains (episodic memory, semantic memory, working memory, perceptual speed, and visuospatial abilities), as shown in Table 1. For each composite score, raw scores on individual tests were converted to *z* scores (based on the mean and SD from baseline) and *z* scores were averaged. A neuropsychologist, blinded to clinical data, reviewed results and summarized impairment. The clinical diagnosis of dementia followed accepted and validated criteria, as recommended by a joint working group, and were made by clinicians with expertise in the evaluation of older persons following review of all clinical data, including cognitive performance, and an in-person examination of each subject (McKhann et al. 1984).

**Table 1:** Cognitive tests used to form cognitive domain scores

Cognitive domain	Cognitive tests
Perceptual speed	Symbol Digit Modalities test; Number Comparison
Episodic memory	Word List Memory, Word List Recall and Word List Recognition from the procedures established by the Consortium to Establish a Registry for Alzheimer’s Disease (CERAD); immediate and delayed recall of Story A from the Logical Memory subtest of the Wechsler Memory Scale-Revised; immediate and delayed recall of the East Boston Story
Semantic memory	Verbal Fluency; an abbreviated version of the Boston Naming Test; an abbreviated version of the National Adult Reading Test
Working memory	Digit Span Forward and Backward of the Wechsler Memory Scale-Revised; Digit Ordering; two indices from a modified version of the Stroop Neuropsychological Test
Visuospatial ability	Items from Judgment of Line Orientation and Standard Progressive Matrices

### MRI data acquisition

Brain MR imaging was conducted on a 1.5 Tesla General Electric (Waukesha, WI) MRI scanner. High-resolution *T*_1_-weighted anatomical data were obtained using a 3D magnetization-prepared rapid acquisition gradient echo (MPRAGE) sequence with the following parameters: echo-time (TE) = 2.8 ms, repetition time (TR) = 6.3 ms, preparation time = 1000 ms, flip angle = 8°, field of view (FOV) = 24 cm × 24 cm, 160 sagittal slices, 1 mm slice thickness, no gap, 224 × 192 acquisition matrix reconstructed to a 256 × 256 image matrix, and two repetitions. *T*_2_-weighted fluid-attenuated inversion recovery (FLAIR) data were collected using a 2D fast spin-echo sequence with the following parameters: TE = 120 ms, TR = 8 s, inversion time = 2 s, FOV = 24 cm × 24 cm, 42 oblique axial slices, slice thickness = 3 mm, no gap, 256 × 224 acquisition matrix reconstructed to a 256 × 256 image matrix.

### Image processing

MRI data were automatically segmented to generate total gray matter and total WMH volume data. First, the two copies of *T*_1_-weighted MPRAGE data collected on each participant were spatially co-registered and averaged. Gray matter was automatically segmented using FreeSurfer (http://surfer.nmr.mgh.harvard.edu), as recently described elsewhere (Fleischman et al. 2014). The total gray matter volume was then measured for each participant and normalized by the corresponding intracranial volume (ICV) generated by FreeSurfer, by dividing by the corresponding ICV, as previously described (Fleischman et al. 2014). The total gray matter volume variable used in the analyses included cortical, subcortical, and other gray matter regions (including the cerebellum).

The average *T*_1_-weighted MPRAGE data for each participant was registered to the corresponding *T*_2_-weighted FLAIR data using affine registration (FLIRT, FMRIB, University of Oxford, UK) (Smith et al. 2004). The brain was extracted from the co-registered MPRAGE and FLAIR image volumes (BET, FMRIB, University of Oxford, UK) (Smith 2002). WMH lesions were then automatically segmented for each participant using a support vector machine classifier considering both *T*_1_-weighted MPRAGE and *T*_2_-weighted FLAIR information (WMLS, SBIA, University of Pennsylvania, PA) (Zacharaki et al. 2008). Maps of WMH were generated. The total volume of brain tissue affected by WMH was measured for each participant and then divided by the corresponding ICV.

For each subject, the average *T*_1_-weighted MPRAGE data in FLAIR space were non-linearly registered to those of all other participants using the Automatic Registration Toolbox (ART) (Ardekani et al. 2005). The resulting ART transformations were averaged and applied to the map of WMH of the subject. The same approach was followed for all other subjects, transforming all WMH maps to population space for the purposes of voxel-wise analysis.

### Analytic approach

We first examined basic statistical characteristics of WMH data, including distribution and outliers. Given the skewed distribution of the total WMH volumes, the values (which were first divided by ICV) were logarithmically transformed (base 10), a transformation employed by others (Gunning-Dixon and Raz 2003; Jeerakathil et al. 2004; Hedden et al. 2012).

All subsequent analyses adjusted for age (at the time of the MRI), sex, and education. The first set of analyses examined the relation of total WMH volume with level of cognition. To do this, we constructed a set of five multiple linear regression models with each of the cognitive domains scores as separate outcomes. Secondary analyses were then conducted to examine the relative effects of WMH on cognition, compared to that of basic demographic variables, and explore whether other factors (e.g., age) modify associations of total WMH volume with cognition.

The second set of analyses examined the relation of WMH volume and gray matter volume to cognition. First, we conducted linear regression analyses with the cognitive domains as separate outcomes, and included terms for age, sex, education, and gray matter volumes. Next, we obtained the partial correlation of the two MRI volumes controlling for age, sex, and education. We then constructed a set of analyses including both MRI measures in the models, to examine for independence of the relation of WMH volume and gray matter volume to cognition. We have used this analytic approach in previous studies to examine for independence of effects and potential sequence of events (Bennett et al. 2003, 2004). Analyses were programmed in SAS version 9.3 (SAS Institute Inc, Cary, NC), and models were validated graphically and analytically.

In addition, voxel-wise analysis was conducted for those cognitive domains that were significantly associated with total WMH volume, to identify those brain regions in which presence of WMH was associated with lower cognition. Voxel-wise multiple linear regressions were adjusted for age, sex, education, and total gray matter volume. The null distribution was built using the ‘‘randomise’’ tool in FSL (FMRIB, University of Oxford, UK) and 5000 permutations of the data. Differences were considered significant at *p* < 0.05, family wise error (FWE) corrected. The threshold-free cluster enhancement (TFCE) method was used to define clusters with significant differences (Smith and Nichols 2009).

## Results

### Subjects

There were 1740 women and men enrolled in the Memory and Aging Project, of whom 1683 had completed the baseline evaluation when the data were extracted for this study. Of these, 462 had a brain MRI scan, and 423 of these scans (92 %) passed quality control. *T*_1_-weighted MPRAGE and *T*_2_-weighted FLAIR data were processed on the first 333 scans. After excluding 119 subjects with MCI and another 5 subjects with dementia identified at or before the year of the MRI, there were 209 subjects without overt cognitive impairment remaining who were included in analyses for this study. Demographic, clinical, and radiographic characteristics of these individuals are described in Table 2.

**Table 2:** Subject characteristics

Characteristic^a^	*n* = 209
Age, years	80.2 (7.2)
Female, *n* (%)	154 (74 %)
Education, years	15.2 (2.9)
Mini-Mental State Examination (MMSE) score, /30	28.9 (1.2)
Perceptual speed score	0.371 (0.642)
Episodic memory score	0.602 (0.494)
Semantic memory score	0.438 (0.526)
Working memory score	0.335 (0.648)
Visuospatial ability score	0.420 (0.594)
Total gray matter volume, (tenths of % ICV)	339.6 (41.4)
Total WMH volume (% ICV)^b^	0.89 % (0.91 %)
Total WMH volume (% ICV), after logarithm^c^	−0.24 (0.40)

^a^Mean (SD), unless otherwise specified

^b^ *WMH* white matter hyperintensities; values expressed as  % of intracranial volume

^c^Logarithm, base 10; used in regression models

### WMH and age

**Fig. 1:**
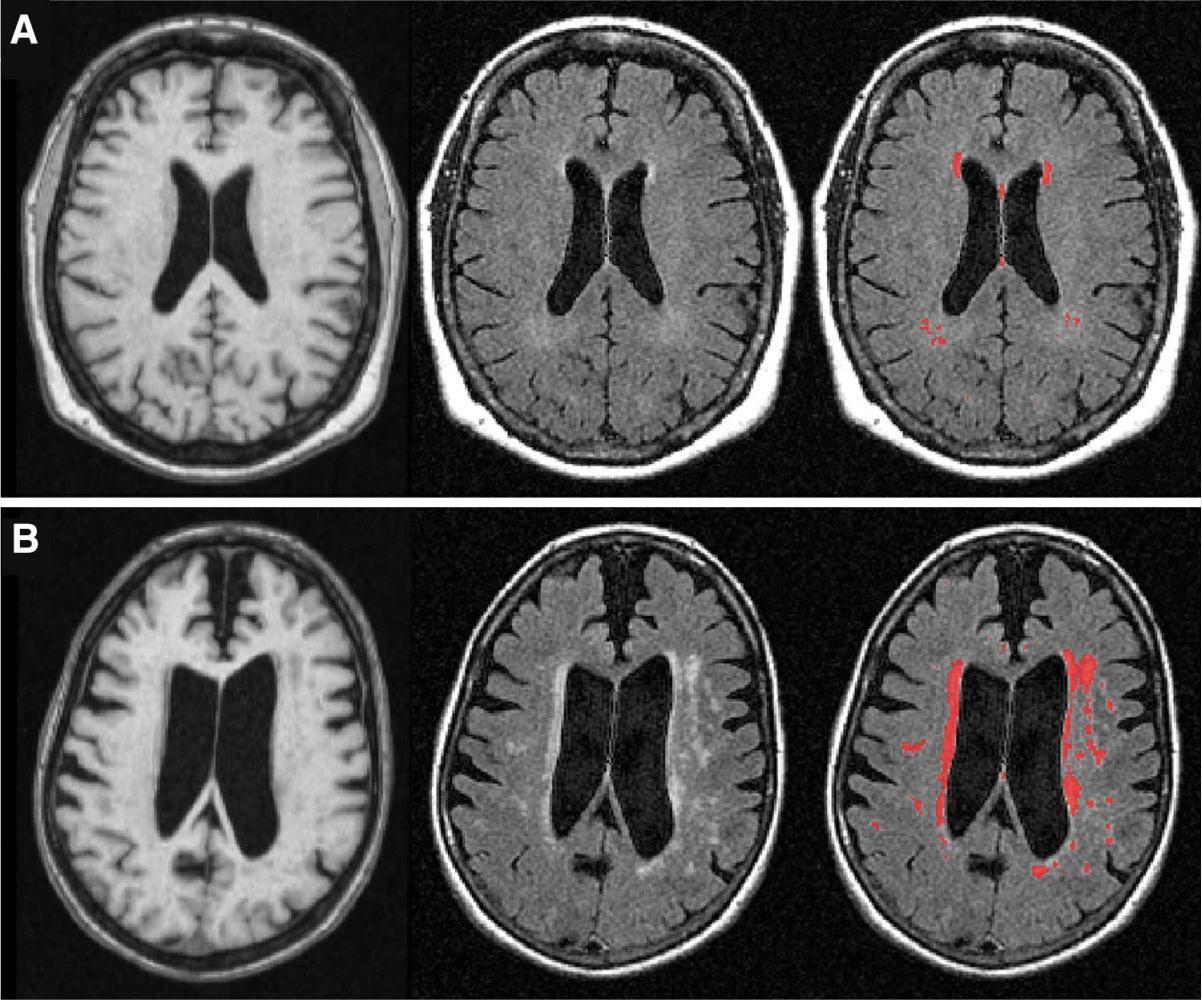
Brain MRI images from two individuals, one with small (**a**), and one with large (**b**) total WMH volume. *Top row* (**a)** shows one individual with small (25 %) total WMH volume, and *bottom row* (**b)** shows another individual with large (75 %) total WMH volume. The *left column* shows averaged *T*_1_-weighted MPRAGE images reformatted from sagittal to axial plane, the *middle column* shows raw axial *T*_2_-weighted FLAIR images, and the *right column* shows in *red* the corresponding WMH lesions segmented automatically based on *T*_1_- and *T*_2_-weighted signals

All subjects in this study of older community-dwelling individuals without dementia or MCI (mean age 80 years, range 60–100 years), had some WMH detected on brain MRI (mean 0.89 %-ICV, SD 0.91 %-ICV). The WMH volumes quantified ranged from 0.05 to 5.04 % of ICV, with the largest value more than 100 times the smallest value. Figure 1 shows images from individuals with WMH at the first and third quartiles of volume. The coefficient of variation of WMH volume data (values expressed as % of ICV) was 103 %, and the skewness coefficient was 2.0. To improve the suitability of regression methods, WMH as % of ICV was transformed using the base 10 logarithm. The mean value of the transformed data was −0.24 units (SD 0.40), and the distribution was less skewed (skewness = 0.2). All subsequent analyses used this transformation of total WMH volume. A Spearman correlation showed that larger WMH volumes were associated with increased age (*r*_s_ = 0.41; *p* < 0.001).

### Total WMH volume and cognition

In the first set of analyses, we examined the relation of total WMH volume to level of cognitive function, in linear regression models adjusted for age, sex, and education. We found that larger (smaller) WMH volumes were associated with lower (higher) perceptual speed scores (*p* < 0.001), but not with levels in the four other cognitive domains, episodic memory, semantic memory, working memory, or visuospatial abilities (all *p* > 0.10), as shown in Table 3.

**Table 3:** Relationship of total WMH volume to five cognitive domains

Composite score of cognition	Estimate	SE	*p* value
Perceptual speed	−0.462	0.110	<0.001
Episodic memory	−0.102	0.089	0.255
Semantic memory	−0.145	0.090	0.109
Working memory	0.032	0.122	0.792
Visuospatial ability	−0.062	0.103	0.543

Estimates are coefficient of log10 (total WMH volume) in linear regression models adjusted for age, sex, and education

We next wished to assess the magnitude of the effect of WMH on cognition. To do so, we compared the effect of total WMH volume on perceptual speed to effects of demographic variables. We constructed two models with perceptual speed as the outcome measure: one model with three demographic terms (age, sex, and education), and the other with an additional term for total WMH volume (Table 4). As shown in model 2 of Table 4, after accounting for the demographic variables of age, sex, and education, total WMH volume explained an additional 6 % of the variance in perceptual speed (20–14 = 6). This represents nearly half (6/14, or 43 %) of the variance explained by demographic variables in this sample.

**Table 4:** Estimated perceptual speed, with and without a term for total WMH volume

Variable	Model 1	Model 2
Adjusted *R* ^2^	0.14	0.20
Age	−0.024 (0.006), <0.001	−0.013 (0.006), 0.040
Sex	−0.040 (0.097), 0.677	−0.110 (0.094), 0.245
Education	0. 055 (0.015), <0.001	0.054 (0.014), <0.001
WMH volume	–	−0.462 (0.110), <0.001

Adjusted *R* ^2^ is given as a fraction. Other values are estimate (SE), *p* value

Because age is associated with changes in white matter and cognitive performance, including in cognitively normal adults (Westlye et al. 2012), we conducted additional analyses to examine whether age modifies the relation of total WMH volume to the cognitive domains. In linear regression models with terms for age, sex, and education, and the additional interaction term of WMH × age, we did not find evidence for any interaction (all *p* > 0.33), suggesting that the effect of total WMH volume on cognitive domains is independent of age.

### Total WMH and gray matter volumes, and cognition

The association of total WMH volume with total gray matter volume was first examined, using a Pearson partial correlation of the two volumes controlling for age, sex, and education. We found an inverse correlation of WMH with gray matter, such that larger total WMH volume was correlated with smaller total gray matter volume (*r* = −0.28; *p* < 0.001).

**Fig. 2:**
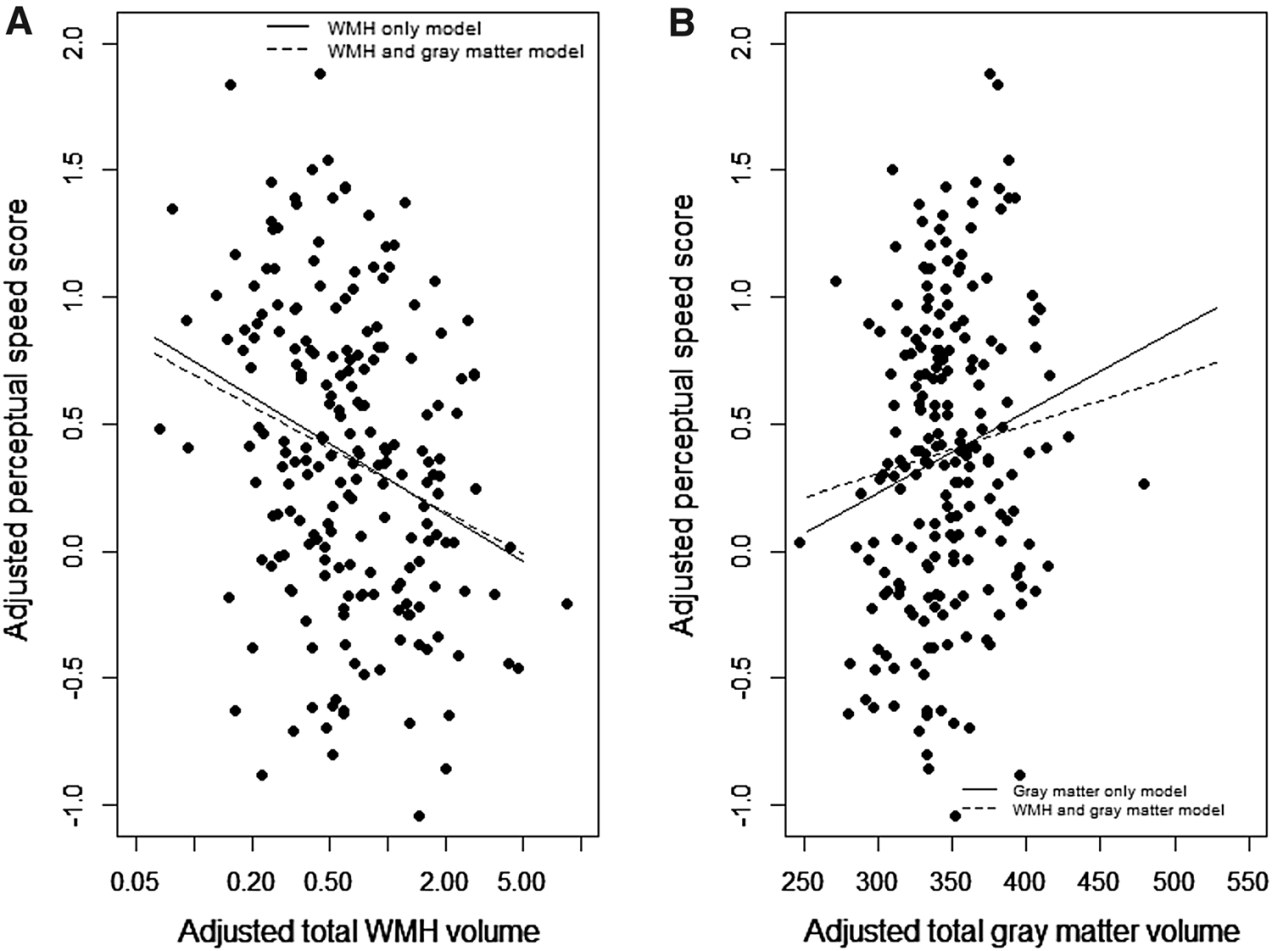
Relationships of total WMH and gray matter volumes to perceptual speed. *Left* *panel* (**a**) level of perceptual speed as a function of total WMH volume (on a geometric axis) in a linear regression model without controlling for total gray matter volume (WMH only model) and in a model controlling for total gray matter volume (WMH and gray matter model). *Right panel* (**b**) level of perceptual speed as a function of total gray matter volume in a linear regression model without controlling for total WMH volume (gray matter only model) and in a model controlling for total WMH volume (WMH and gray matter model). All models and data values are adjusted for age, sex, and education. WMH volumes were analyzed and adjusted in log-10 scale

Next, we examined whether total WMH volume and total gray matter volume had independent associations with cognition. We first examined the relation of total gray matter volume to the five cognitive domains, in multiple linear regression analyses adjusted for age, sex, and education. In these models, smaller total gray matter volumes were associated with lower levels of perceptual speed and episodic memory, but not with the other three cognitive domains (Table 5). Because both MRI measures (total WMH and gray matter volumes) were related to lower perceptual speed, we were next able to test whether the MRI measures had independent associations with perceptual speed. To do this, we created an additional model with terms for both MRI measures included (in addition to age, sex, and education). We found that the association of total WMH volume with perceptual speed was essentially unchanged (compared to the model without the term for gray matter volume) and remained significant, while the association of total gray matter volume with perceptual speed was reduced and no longer significant (Table 6). Figure 2 illustrates the relationship of total WMH and gray matter volumes to perceptual speed. In panel A, the solid line shows the relationship between total WMH volume and level of perceptual speed from a model adjusted for age, sex, and education. Larger WMH volumes are associated with lower levels of perceptual speed. The dotted line represents the relationship after including a term for total gray matter volume. Note that the dotted line is nearly parallel to the solid line, suggesting that gray matter volume does not affect the relationship between WMH volume and cognition (which remains essentially unchanged). In panel B, the solid line shows the relationship of total gray matter volume with level of perceptual speed, with smaller gray matter volumes being associated with lower perceptual speed. The dotted line represents the relationship after including a term for total WMH volume. Note that the dotted line has a less pronounced slope than the solid line, suggesting that the relationship between gray matter volume and cognition is reduced when accounting for WMH volume. In a separate analysis, there was no evidence for an interaction of total WMH and gray matter volumes (*p* = 0.93). Taken together, these results suggest that the association of increased total WMH volume with lower perceptual speed is independent of total gray matter volume.

**Table 5:** Relationship of total gray matter volume to five cognitive domains

Composite score of cognition	Estimate	SE	*p* value
Perceptual speed	0.003	0.001	0.013
Episodic memory	0.003	0.001	0.001
Semantic memory	0.001	0.001	0.237
Working memory	0.001	0.001	0.631
Visuospatial ability	0.002	0.001	0.143

All models adjusted for age, sex, and education

**Table 6:** Relationship of total WMH and gray matter volumes to perceptual speed

MRI measure	Model 1	Model 2	Model 3
	Estimate (SE), *p*	Estimate (SE), *p*	Estimate (SE), *p*
Adjusted *R* ^2^	0.20	0.16	0.21
WMH volume	−0.462 (0.110), <0.001	–	−0.452 (0.114), <0.001
Gray matter volume	–	0.003 (0.001), 0.013	0.002 (0.001), 0.138

All models adjusted for age, sex, and education

*Model 1* WMH volume only, *Model 2* gray matter volume only, *Model 3* both WMH and gray matter volumes

–, term not included in model

**Fig. 3:**
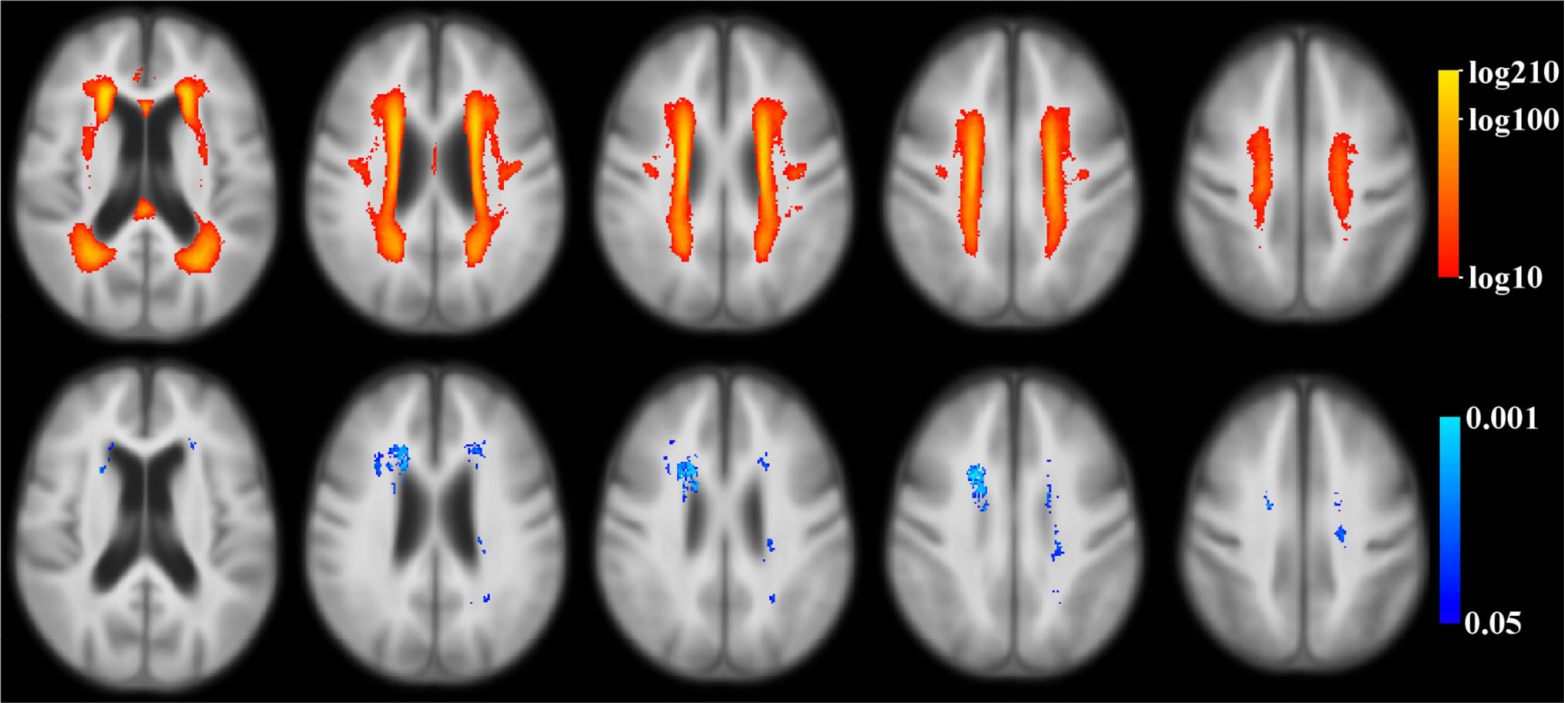
Relationships of WMH to perceptual speed. *Top row* maps of the number of participants with WMH in the same location (*red–yellow* *color scale*, a *logarithmic color scale* is used). Voxels with WMH in fewer than 10 participants are not shown in *color*. *Bottom row* voxels where presence of WMH was associated with lower perceptual speed score, controlling for age, sex, education, and total gray matter volume (*blue color* *scale*)

We also assessed brain voxels in which the presence of WMH was associated with a lower perceptual speed score. Using voxel-wise multiple linear regression analyses controlling for total gray matter volume, we found that the presence of WMH in a number of periventricular areas was associated with a lower perceptual speed score (Fig. 3). These areas included portions of the anterior thalamic radiations, the corpus callosum, and the corona radiata.

## Discussion

In this study of more than 200 older individuals without dementia or mild cognitive impairment, we found that WMH on brain MRI were common, associated with older age, and related to cognition. Specifically, larger total WMH volumes were associated with lower levels of perceptual speed, but not with memory or other cognitive functions. The effect of total WMH volume on perceptual speed was nearly half that of the demographic variables combined, and the association of total WMH volume with perceptual speed was independent of age. Notably, when considering total WMH and gray matter simultaneously, we found that the association of larger total WMH volume with lower perceptual speed in older individuals without overt cognitive impairment was independent of total gray matter volume.

It is now well recognized that WMH are commonly observed in the brains of older persons, and that the burden of WMH increases with age (Breteler et al. 1994; Ylikoski et al. 1995; Gouw et al. 2008; Raz et al. 2012). This burden may be, in part, due to increased neurologic diseases in aging, including cerebrovascular disease (Burton et al. 2004; Rost et al. 2010). Yet, WMH are also common in middle-aged healthy adults (with one study reporting 51 % of 428 healthy subjects in their 40’s having WMH), and in older adults without known neurologic diseases or conditions (Wen et al. 2009; Silbert et al. 2008). Findings from our study are consistent with this literature. With a mean age of about 80 years and no clinically recognized cognitive impairment, all of the subjects in this study had some WMH, mostly in relatively small volumes, as would be expected in a healthy volunteer cohort.

A large body of literature shows that WMH have detrimental effects on neurologic function, including cognition (Silbert et al. 2008, 2012; Debette and Markus 2010; Wakefield et al. 2010; Kloppenborg et al. 2014). WMH have been associated with cognition among persons with a range of cognitive function including dementia, and clinical diagnoses including Alzheimer’s disease (Aggarwal et al. 2010; Smith et al. 2011; Lo et al. 2012; Carmichael et al. 2012; Maillard et al. 2012). Associations with cognition among persons with mild cognitive impairment have also been reported (Yoshita et al. 2006; Debette et al. 2007; Carmichael et al. 2010; Lo et al. 2012). However, little is known about WMH and cognition among persons without dementia (Aggarwal et al. 2010), and even less so among persons without dementia or mild cognitive impairment. Several factors may contribute to the limited knowledge in this area. Some studies have evaluated cognition in seemingly healthy older individuals, but have not excluded persons with mild cognitive dysfunction (Au et al. 2006; Wright et al. 2008; Ishikawa et al. 2012). Other studies identified cognitive impairment but employed a non-specific screening test (e.g., MMSE < 24), which does not exclude all cases with mild dementia or mild cognitive impairment (Söderlund et al. 2006; van den Heuvel et al. 2006; Wakefield et al. 2010). Finally, some studies used only a single measure of cognition or had a small sample (e.g., <50 subjects) (van der Flier et al. 2005; Silbert et al. 2009; Raji et al. 2012).

A few larger studies of WMH and cognition in the elderly, specifically excluding persons with dementia and mild cognitive impairment, have examined the association of WMH with different cognitive domains. In one study, WMH burden was related to performance on timed tasks (Vannorsdall et al. 2009). Two subsequent studies showed an association of WMH with lower executive function but not memory (Murray et al. 2010; Hedden et al. 2012). Here, we found that larger total WMH volume was related to lower scores on a composite measure of perceptual speed. Our finding contributes to the mounting evidence showing that greater WMH is associated with lower cognition, and lower perceptual speed in particular, in individuals without overt cognitive impairment. Further, while the effect of WMH on cognition may be comparable to that of age as suggested by one study (Hedden et al. 2012), we found that WMH volume accounted for nearly half of the variance in perceptual speed explained by demographic variables including age, underscoring the strength of the effect of WMH on cognition in older individuals without overt cognitive impairment.

Factors accounting for the relation of WMH to cognition remain to be elucidated. As increasing age is associated with WMH and also with cognitive impairment, we considered the role of age in our study. All analyses controlled for age, yet we found an association of larger WMH volume with lower cognition, suggesting that age is not a crucial confounder in this relationship. Furthermore, we found no evidence of modification by age of the association between WMH and cognition, suggesting that the relation of WMH to cognition is independent of age. Additional research is needed that examines other factors which may modify the relationship of WMH with cognition among overtly healthy older individuals. Plausible factors include genetic factors, vascular risk factors and diseases (such as hypertension and diabetes), inflammation, and oxidative stress (Novak et al. 2006; Wright et al. 2009; Xu et al. 2010; Satizabal et al. 2012; Raz et al. 2012).

Few studies have examined effects of both WMH and gray matter volumes on different cognitive domains. We are aware of only one other study which considered both total WMH and total gray matter volumes that included persons with normal cognition (He et al. 2012). In this clinic-based study of persons with a range of cognitive function (47 with dementia, 65 with MCI, and 97 with normal cognition), WMH and gray matter volume were separately associated with episodic memory, but in analyses taking both MRI measures into account simultaneously, only gray matter remained associated (He et al. 2012). The authors concluded that gray matter is more closely related to cognition than WMH. A more recent study included exclusively persons without dementia (*n* = 81, all with MMSE ≥ 24), and considered volume in a specific gray matter region (the hippocampus), in addition to WMH (Papp et al. 2014). In this study, WMH and hippocampal volumes were independently associated with processing speed when both MRI measures were considered simultaneously (Papp et al. 2014). Thus, while research has been done in persons without dementia as noted above, we are not aware of any other study in older persons that also restricted their study to those without mild cognitive impairment (MCI), and directly examined the effects simultaneously of both total WMH and total gray matter volumes on different cognitive domains. In comparison to the studies by He et al. and Papp et al., we found, in about 200 community-dwelling persons without dementia or MCI, that larger total WMH volume and smaller total gray matter volume were each separately associated with lower perceptual speed, but when taking both MRI measures into account, WMH remained related to perceptual speed while gray matter volume was no longer related. These results suggest that the association of WMH volume with perceptual speed is independent of gray matter volume. Furthermore, the results suggest that the relationship of gray matter volume to cognition may be accounted for, at least in part, by WMH volume. Also, our finding of an association of gray matter volume with episodic memory, similar to the finding by He et al. (2012) suggests that the relation of MRI data to cognition may vary depending on the particular domain of cognition being examined.

We found regional specificity of the association of WMH with cognition, consistent with a recently published study of adults with a lower age on average and high cognitive function (mean age 60 years, and all with MMSE ≥ 25; Birdsill et al. 2014). In that study, authors exploited voxel-wise analyses and found an association of WMH, most notably in the superior corona radiata, with lower cognitive speed and flexibility (Birdsill et al. 2014). Our study extends this finding, by showing that the periventricular brain regions with WMH, including the corona radiata, and also the thalamic radiations and corpus callosum, play a role in the association of WMH with lower perceptual speed, after controlling for total gray matter volume. While the literature has previously suggested that periventricular lesions are associated with impaired perceptual speed, further studies using modern imaging and analytic techniques are needed to examine regional relationships of WMH with cognition (van den Heuvel et al. 2006).

There are several possible interpretations of our results evaluating both WMH and gray matter volumes. First, WMH volume may be a confounder in the relation of gray matter volume with cognition. Yet, the association of gray matter volume with cognition is biologically plausible and extensive literature supports such an association. Second, early pathologic processes may occur in the gray matter and lead to loss of gray matter volume (e.g., neuronal cell body dysfunction and neuronal death, resulting in gray matter atrophy), and may then be followed by changes in the white matter (e.g., changes in axons or the myelin covering the axons, and WMH), and then ultimately lead to brain dysfunction (e.g., lower cognitive function). In this case, WMH would mediate the relationship between gray matter volume and cognition. This sequence of events in the pathophysiologic cascade, in which WMH are more closely related to cognition than gray matter, contrasts with a previously published study (He et al. 2012). The discrepant results may be due to differences in source of participants (e.g., community-based individuals without overt cognitive impairment vs. persons with a range of cognitive function). Indeed, the associations of brain pathology with cognition may not be the same across a spectrum of health/disease. It is possible that once individuals develop cognitive impairment or dementia, that gray matter volumes become a more important factor in the association with cognitive function, compared to WMH. By analogy, we have previously observed differing results in the relation of two Alzheimer’s disease pathology measures (amyloid and neurofibrillary tangles) to cognition, in studies restricted to persons without cognitive impairment vs. not (Bennett et al. 2004, 2012b). In addition, discrepant results regarding the importance of WMH may also be due to differences in study design and methodology, including MRI data acquisition and image processing, neuropsychological test selection, data summary and analyses. Further study is clearly needed to definitively establish the relationship of WMH, gray matter, and cognition. Elucidation of the pathological processes that lead to lower cognitive function, and of sequences of events in that pathway (e.g., whether reduced gray matter volume occurs before WMH, or vice versa), is important to better understand mechanisms of aging and disease, and to inform on targets for intervention to prevent cognitive decline. To our knowledge, this study is the first that has tested independence of total WMH and gray matter volume effects on different cognitive domains in older individuals without overt cognitive impairment.

Weaknesses of this study should be noted. First, our aim was to examine for independent effects of total WMH and total gray matter volumes on cognition, and thus we did not examine relationships in analyses that considered both regional WMH and regional gray matter data. While our study’s novelty is enhanced by voxel-wise analyses of the association of WMH with perceptual speed, further examination of the relation of regional MRI data to cognitive domains is likely to shed additional light on underlying pathophysiologic processes and will need to be explored in subsequent studies. Second, this study presents cross-sectional analyses of the relation of WMH to cognition. Data on the evolution of WMH changes is expected to be more informative and we have plans to analyze the relation of change in WMH to cognition in the future as data become available. Third, while secondary analyses tested for effect modification by age, we did not take other factors, such as vascular factors, into account. We are collecting MRI data on brain infarcts and, as all participants were enrolled in a clinical-pathologic cohort study with a high autopsy rate, neuropathologic data on cerebral infarcts will become available for future analyses. We plan to examine this issue more directly in individuals with clinical, brain MRI, and neuropathologic data.

Strengths of the study are also worth noting. First, analyses were conducted in older community-dwelling women and men who were clinically well-characterized, and did not have dementia or mild cognitive impairment. This allowed for the examination of the relationship of WMH to cognition in a group of older individuals without overt cognitive impairment. Second, detailed neuropsychological evaluations proximate to the MRI scan were conducted, and outcome measures included five different cognitive domains. Each cognitive domain was a composite measure based on two or more individual tests, thus decreasing ceiling effects. Ceiling effects are of particular concern in this study given the high cognitive performance of individuals included in analyses. Finally, several strengths of the study are derived from the methods used to assess and analyze WMH data. Total brain WMH volume data were collected in an automated fashion, removing user bias, and were based on information from both *T*_1_-weighted and FLAIR images, improving robustness of segmentation and lending internal validity to the study. Furthermore, the consideration of WMH as continuous measures of volume of lesions, rather than as categories, increased the power to detect associations with cognition. Finally, we used voxel-wise analyses to examine brain regions in which presence of WMH was associated with lower cognitive function.
